# Progression of motor deficits in glioma-bearing mice: impact of CNF1 therapy at symptomatic stages

**DOI:** 10.18632/oncotarget.15328

**Published:** 2017-02-15

**Authors:** Eleonora Vannini, Federica Maltese, Francesco Olimpico, Alessia Fabbri, Mario Costa, Matteo Caleo, Laura Baroncelli

**Affiliations:** ^1^ Institute of Neuroscience, National Research Council (CNR), Pisa, Italy; ^2^ BioSNS laboratory, Scuola Normale Superiore di Pisa, Pisa, Italy; ^3^ Istituto Superiore di Sanità, Rome, Italy

**Keywords:** glioma, motor cortex, behavioral test, CNF1, mouse model

## Abstract

Glioblastoma (GBM) is the most aggressive type of brain tumor. In this context, animal models represent excellent tools for the early detection and longitudinal mapping of neuronal dysfunction, that are critical in the preclinical validation of new therapeutic strategies. In a mouse glioma model, we developed sensitive behavioral readouts that allow early recognizing and following neurological symptoms. We injected GL261 cells into the primary motor cortex of syngenic mice and we used a battery of behavioral tests to longitudinally monitor the dysfunction induced by tumor growth. Grip strength test revealed an early onset of functional deficit associated to the glioma growth, with a significant forelimb weakness appearing 9 days after tumor inoculation. A later deficit was observed in the rotarod and in the grid walk tasks. Using this model, we found reduced tumor growth and maintenance of behavioral functions following treatment with Cytotoxic Necrotizing Factor 1 (CNF1) at a symptomatic stage. Our data provide a detailed and precise examination of how tumor growth reverberates on the behavioral functions of glioma-bearing mice, providing normative data for the study of therapeutic strategies for glioma treatment. The reduced tumor volume and robust functional sparing observed in CNF1-treated, glioma-bearing mice strengthen the notion that CNF1 delivery is a promising strategy for glioma therapy.

## INTRODUCTION

Gliomas are malignant primary tumors of the central nervous system that arise from astrocytes, oligodendrocytes and their precursors. Glioblastoma (GBM) is the most aggressive type of glioma [1] with a median survival expectancy of 15-18 months since the first diagnosis and a five-year survival rates <10% [2, 3]. The standard of care in GBM patients consists in the surgical resection of tumoral mass followed by cycles of radiotherapy and chemotherapy with temozolomide (TMZ). Unfortunately, this combined intervention protocol is only partly effective [4]. Thus, there is an urgent need to find innovative approaches for the treatment of GBM patients [5]. These novel strategies should aim not only at targeting glioma growth but also at preventing functional deterioration of spared brain networks. Typically, many gliomas in patients become symptomatic with focal neurological deficits (i.e. motor weakness or seizures), but in animal models there are very few tools available for the early detection and longitudinal mapping of neuronal dysfunction. These tools may prove very useful in the preclinical study of potential therapeutic strategies and in the evaluation of side effects of radiotherapy and chemotherapy. Accordingly, our first goal was the development of sensitive behavioral readouts that allow recognizing early neurological symptoms and following motor dysfunction over glioma progression in a mouse model. With these tools in hand, we tested the effectiveness of anti-glioma therapy in mice at symptomatic stages. Specifically, we exploited Cytotoxic Necrotizing Factor 1 (CNF1), a protein produced by *E. coli* leading to the long-lasting activation of intracellular Rho GTPases [6, 7]. We recently showed that CNF1 (i) leads to multinucleation, senescence and death of murine and human glioma cells, increasing the survival of glioma-bearing mice [8, 9], and (ii) spares neuronal responses and architecture in the tissue surrounding the tumor mass [9]. In our previous studies, CNF1 was administered at a very early stage of the disease (i.e., only 5 days after tumor induction); therefore, the evaluation of the effects of treatment at later stages is needed to better understand the translational value of this protein.

Based on these premises, we have set up a mouse model of GBM by injecting GL261 cells [8, 10] into the primary motor cortex in order to monitor longitudinally behavioral dysfunction induced by tumor growth. The primary motor cortex is located in the frontal cortex [11], which is the topographic location showing the densest occurrence of gliomas [12]. In addition, the choice of the motor cortex as injection site was due to the availability of sensitive behavioral tests allowing longitudinal assessment of motor abilities in the same animals. After the characterization of the progressive impairment in motor output, we treated glioma-bearing mice with CNF1 at the symptomatic stage and we evaluated whether Rho GTPase long-lasting activation was effective in reducing glioma volume and in promoting the recovery of associated physiological deficits. In this paper, we demonstrate reduced tumor growth and maintenance of behavioural functions following treatment with CNF1 at a symptomatic stage.

## RESULTS

### Longitudinal progression of motor deficits in a mouse model of high-grade glioma

**Figure 1:**
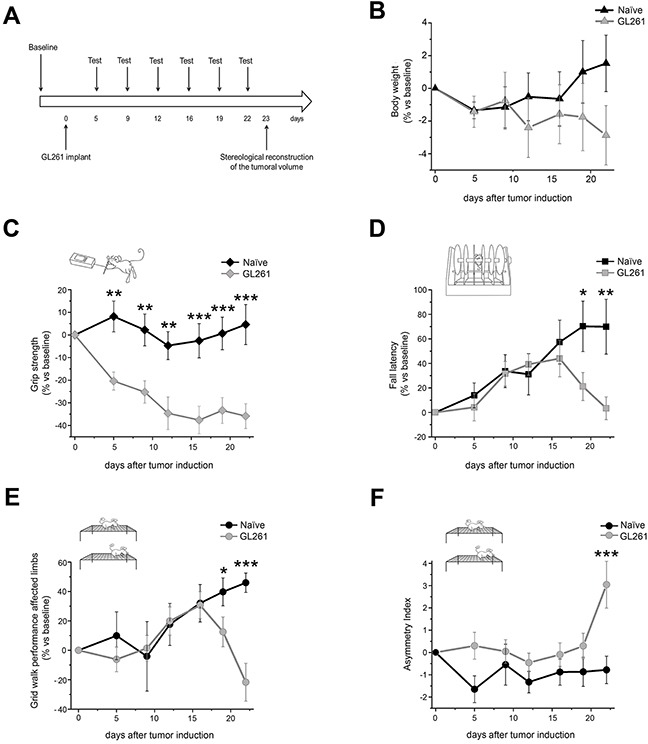
Behavioral characterization of glioma-bearing mice **A**. Experimental protocol used for the characterization of the GBM model in primary motor cortex. Glioma-bearing animals were tested before (baseline) and 5, 9, 12, 16, 19 and 22 days after tumor induction. Control naïve animals were tested in the same days. Moreover, 23 days after GL261 cells implant, the tumor volume was stereologically reconstructed and measured. **B**. Body weight assessment in both control naïve animals (black line, n = 11) and glioma-bearing mice (grey line, n = 9). No significant difference was detected between the two experimental groups (Two-way RM ANOVA on rank transformed data, effect of treatment p = 0.611, treatment x day interaction p = 0.372). **C**. Grip strength test. The performance of glioma-bearing mice (grey line, n = 9) displayed a significant difference with respect to naïve starting from day 5 (black line, n = 11; Two-way RM ANOVA, treatment x day interaction p < 0.01, post hoc Holm-Sidak method for day 5, 9, 12 p < 0.01, for day 16, 19, 22 p < 0.001). **D**. Rotarod test. A significant difference between glioma-bearing mice (grey line, n = 9) and control animals (black line, n = 11) was found 19 and 22 days after GL261 cells injection (Two-way RM ANOVA on rank transformed data, treatment x day interaction p < 0.01, post hoc Holm-Sidak method; for day 19 p < 0.05, for day 22 p < 0.01). **E, F**. Grid walk test. A significant difference in the number of correct steps performed with affected limbs was evident between the two groups (grey line, n = 9 for glioma-bearing mice; black line, n = 11 for controls) 19 and 22 days after GL261 cell injection (Two-way RM ANOVA on rank transformed data, treatment x day interaction p < 0.01, post hoc Holm-Sidak method, for day 19 p < 0.05, for day 22 p < 0.001; panel E). Statistical analysis of asymmetry index revealed a significant difference between control naïve animals and glioma-bearing mice at day 22 (Two-way RM ANOVA on rank transformed data, treatment x day interaction p < 0.05, post hoc Holm-Sidak method, p < 0.001; panel F). Data are expressed as mean ± SEM. * p <0.05, ** p < 0.01, *** p <0.001.

GL261 cells were stereotaxically injected into the forelimb representation in mouse primary motor cortex [11, 13]. To behaviorally characterize our glioma model we used an experimental protocol (Figure 1A), which allowed us to longitudinally monitor animals’ performance during the progression of the disease and to evaluate the temporal profile of symptom appearance. We first assessed whether tumor growth caused a systemic weakening of organism physiology and function within the time window of behavioral testing by measuring changes in animals’ body weight. We found only a slight weight reduction in glioma-bearing mice (n = 9) starting from day 19, and overall no significant difference was detected with respect to the naïve control group (n = 11; Two-way RM ANOVA on rank transformed data, effect of treatment p = 0.611, treatment x day interaction p = 0.372; Figure 1B).

On the other hand, specific behavioral tests were effective in detecting limb dysfunction. The grip strength test showed a very precocious difference between naïve and glioma-bearing mice: indeed, 5 days after the tumor induction the force exerted by glioma-bearing animals was significantly decreased with respect to that measured for controls (Two-way RM ANOVA, effect of treatment p < 0.001, post hoc Holm-Sidak method, p < 0.01; Figure 1C). Importantly, this difference was maintained for all the subsequent days with glioma-bearing mice showing a progressive deterioration of forelimb strength until the day 12 (Two-way RM ANOVA, treatment x day interaction p < 0.01, post hoc Holm-Sidak method for day 5, 9, 12 p < 0.01, for day 16, 19, 22 p < 0.001; Figure 1C). To understand whether the early deficit in strength was partly due to the lesion induced by surgical procedure, we simulated GL261 implant (by injecting vehicle solution) in a different group of animals (sham group), which were tested 5 and 9 days after surgery. We detected a small difference in grip strength between sham and naïve animals at day 5 (Two-way RM ANOVA, treatment x day interaction p < 0.05, post hoc Holm Sidak method, p < 0.05; Supplementary Figure 1), which completely recovered at day 9 (p = 0.231; Supplementary Figure 1). These results indicate that the initial decrease in grip strength is partly related to surgery-dependent weakness, while the motor deficit is totally ascribable to the presence of tumor mass starting from day 9.

In the rotarod test the onset of behavioral deficit was delayed with respect to that observed in the grip strength task. It is worth noting that in this test a consistent learning component influences the animals’ performance, with a significant improvement through successive trials. Indeed, we observed that both experimental groups showed an improvement in their capability to walk on the rotating drum in the first days of testing. However, while control animals continued to improve their motor skills throughout the experimental protocol, glioma-bearing mice abruptly stopped their learning at day 12 followed by decay in performance (Figure 1D). Quantification of relative fall latency in glioma-bearing and control mice revealed a significant difference in the motor performance 19 and 22 days after GL261 cells injection (Two-way RM ANOVA on rank transformed data, treatment x day interaction p < 0.01, post hoc Holm-Sidak method; for day 19 p < 0.05, for day 22 p < 0.01; Figure 1D).

The grid walk task confirmed the results obtained in the rotarod test. Also in this case we noticed the influence of learning in animals’ performance. Task practice, in fact, led to a reduction in the percentage of errors made with affected limbs in both experimental groups starting from day 9. While in naïve control animals a steady improvement in motor performance was maintained, glioma-bearing mice decreased the number of correct steps starting from day 19 (Figure 1E). Quantification of errors made with affected limbs in glioma and control mice detected a significant difference in animals’ performance 19 and 22 days after GL261 cells injection (Two-way RM ANOVA on rank transformed data, treatment x day interaction p < 0.01, post hoc Holm-Sidak method, for day 19 p < 0.05, for day 22 p < 0.001; Figure 1E). On the contrary, no significant difference between the two groups emerged in the number of incorrect steps performed with unaffected limbs (Two-way RM ANOVA on rank transformed data, effect of treatment p = 0.969, treatment x day interaction p = 0.870; Supplementary Figure 2A). Consistently, the asymmetry index (calculated by subtracting the percentage of errors made with affected limbs to that of unaffected limbs) was increased in glioma-bearing animals. Statistical analysis revealed a significant difference in the asymmetry index between naïve and glioma-bearing mice at day 22 (Two-way RM ANOVA on rank transformed data, treatment x day interaction p < 0.05, post hoc Holm-Sidak method, p < 0.001; Figure 1F), suggesting that the performance at the last day of our experimental protocol was the most predictive of tumor mass growth. No difference was found between the two groups for the total number of steps (Two-way RM ANOVA on rank transformed data, effect of treatment p = 0.564, treatment x day interaction p = 0.870; Supplementary Figure 2B).

### Tumor growth in the motor cortex

To determine tumor growth rate in the motor cortex, we carried out a stereological reconstruction of the tumor mass in the same mice used for behavioral assessment. Animals were sacrificed 23 days after the inoculation of GL261 cells (Figure 1A). The histological analysis showed an evident tumorous mass (Supplementary Figure 3A), the size of which was totally comparable to that measured in mice subjected to GL261 cells injection into the visual cortex (Mann-Whitney test; p = 0.954; [9]). We also compared the tumor volume measured at 14 (n = 6) and 23 days (n = 9) after GL261 cell implant (Supplementary Figure 3B). As depicted in Supplementary Figure 3B, we found that the tumor mass was already in the order of few mm^3^ at 14 days, i.e. the size of the entire primary forelimb motor area in mice [11]. Then, tumor dramatically grew from 14 to 23 days.

### Late CNF1 treatment prevents functional deterioration of motor capabilities in glioma-bearing mice

**Figure 2:**
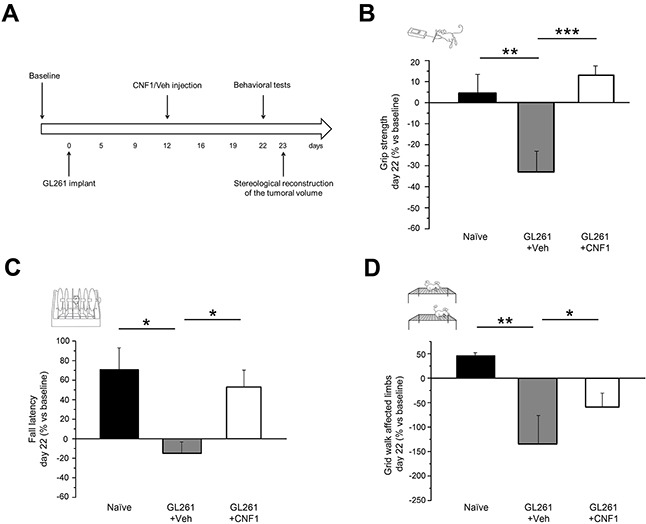
Preservation of motor capabilities in CNF1 glioma-bearing mice **A**. Experimental protocol. CNF1/Veh treatment was performed 12 days after tumor implantation and glioma-bearing animals were tested with grip strength, rotarod and grid walk tasks before (baseline) and 22 days after the tumor induction. The stereological tumor reconstruction was performed at day 23. **B**. Grip strength test. CNF1 glioma-bearing mice (white column, n = 12) exerted with their forelimbs an average force significantly higher than vehicle-treated animals (grey column, n = 9; One-way ANOVA, post hoc Holm Sidak test, p < 0.001) and comparable with that of naïve mice (black column, n = 11; One-way ANOVA, post hoc Holm Sidak test, p = 0.423). **C**. Rotarod test. Fall latency of CNF1 glioma-bearing mice (white column, n = 12) was significantly longer with respect to vehicle-treated animals (grey column, n = 9; One-way ANOVA on ranks, post hoc Dunn's test, p < 0.05) and comparable to naïve mice (black column, n = 11; One-way ANOVA, post hoc Dunn's test, p > 0.05). **D**. Grid walk test. The number of errors performed with affected limbs in the grid walk was strongly reduced in CNF1 (white column, n = 12) as compared to vehicle (grey column, n = 9) glioma-bearing mice (One-way ANOVA, post hoc Holm Sidak test, p < 0.05). The protective effects of CNF1 were not complete as glioma-bearing mice treated with CNF1 displayed a clear trend toward an increased number of errors with respect to naïve controls (One-way ANOVA, post hoc Holm Sidak test, p = 0.08). Data are expressed as mean ± SEM. * p <0.05, ** p < 0.01, *** p <0.001.

On the basis of the behavioral characterization, we delivered CNF1 following the appearance of initial clinical signs of tumor growth. To evaluate CNF1 effects on motor capabilities of glioma-bearing mice, the behavioral phenotype of both CNF1 and Veh-treated animals was analyzed 22 days after GL261 cells injection using grip strength, rotarod and grid walk tests (Figure 2A). We found that the performance of CNF1-treated mice (CNF1, n = 12) was significantly improved with respect to vehicle-treated animals (Veh, n = 9) both in the grip strength test (One-way ANOVA, post hoc Holm-Sidak method, p < 0.001, Figure 2B) and in the rotarod task (One-way ANOVA on ranks, post hoc Dunn's method, p < 0.05, Figure 2C). Importantly, grip strength (One-way ANOVA, post hoc Holm-Sidak method, p = 0.423) and fall latency (One-way ANOVA on ranks, post hoc Dunn's method, p > 0.05) measured in CNF1-treated mice were totally comparable to those recorded from naïve animals (Figure 2B, 2C). In addition, the number of errors made with affected limbs in the grid walk test was strongly reduced in CNF1 compared to Veh-treated, glioma-bearing mice (One-way ANOVA, post hoc Holm-Sidak method, p < 0.05, Figure 2D). Despite no significant difference was detected between naïve, Veh- and CNF1-treated glioma-bearing mice in the number of errors made with unaffected limbs (One-way ANOVA on ranks, p = 0.649), it is also worth noticing that CNF1 mice exhibited a trend towards a better performance with respect to the other two groups of animals (Supplementary Figure 4).

**Figure 3:**
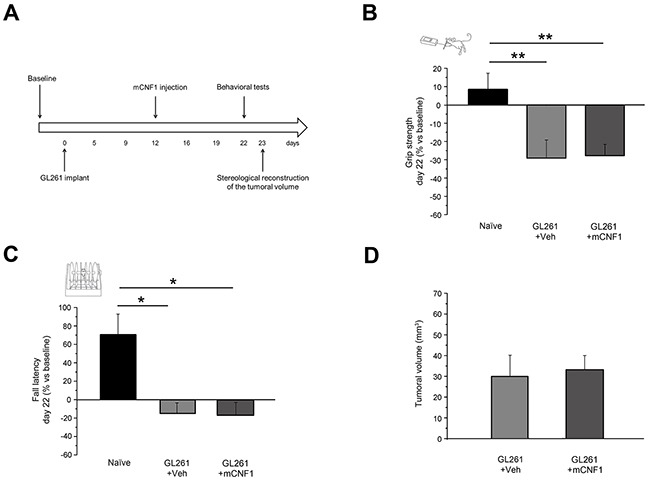
Mutated CNF1 (mCNF1) did not exert any beneficial effect on motor capabilities of glioma-bearing mice **A**. Experimental protocol. mCNF1 treatment was performed 12 days after tumor implantation and glioma-bearing animals were tested with grip strength and rotarod tasks before (baseline) and 22 days after the tumor induction. The stereological tumor reconstruction was performed at day 23. **B**. Grip strength of mCNF1 glioma-bearing mice (dark grey column, n = 11) was significantly lower than in naïve (black column; One-way ANOVA, post hoc Holm Sidak test, p < 0.01) and comparable with that of vehicle-treated animals (grey column; p = 0.910). **C**. Fall latency of mCNF1 glioma-bearing mice was significantly shorter with respect to naïve (One-way ANOVA on ranks, post hoc Dunn's method, p < 0.05) and comparable to Veh-treated mice (p > 0.05). **D**. Stereological reconstruction revealed that mCNF1 treatment was not able to reduce tumor volume (t-test, p = 0.781 vs. Veh group). Data are expressed as mean ± SEM. * p <0.05, ** p < 0.01.

As a further control, we evaluated the effects of the late delivery (12 days after GL261 implantation) of a recombinant form of CNF1 (mCNF1) that lacks enzymatic activity due to a single aminoacid change [14, 15]. We found that motor capabilities of mCNF1-treated mice (mCNF1, n = 11) were comparable to those of vehicle-treated animals in the grip strength test (One-way ANOVA, post hoc Holm-Sidak method, p = 0.91, Figure 3B) and in the rotarod task (One-way ANOVA on ranks, post hoc Dunn's method, Figure 3C). Grip strength (One-way ANOVA, post hoc Holm-Sidak method, p < 0.01) and fall latency (One-way ANOVA on ranks, post hoc Dunn's method, p < 0.05) measured in mCNF1-treated mice were significantly impaired with respect to naïve animals.

In summary, all the behavioral tests showed that glioma-bearing mice treated with CNF1 displayed a significant preservation of motor performance, demonstrating that the delivery of this toxin prevents physiological deficits associated with glioma growth, even at a symptomatic stage of the disease.

### Tumor size is reduced in CNF1-treated mice

**Figure 4:**
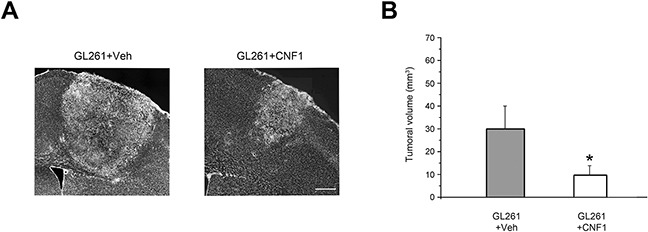
Glioma volume reduction after CNF1 treatment in glioma-bearing mice **A**. Representative images taken from a vehicle and a CNF1 glioma-bearing mouse 23 days after tumor implantation. Scale bar = 150 μm. **B**. Stereological reconstruction in both vehicle (grey column, n = 8) and CNF1 (white column, n = 12) glioma-bearing mice revealed that CNF1 treatment reduced tumor volume (Mann-Whitney Rank Sum Test, p < 0.05). Data are expressed as mean ± SEM. * p <0.05.

The functional recovery observed in CNF1-treated mice strongly suggested that toxin administration significantly impacted glioma growth. Indeed, the stereological reconstruction of tumor volume at day 23 revealed a significantly smaller tumor mass in glioma-bearing animals treated with CNF1 with respect to Veh-treated mice (Mann-Withney Rank Sum test, p < 0.05; Figure 4A, 4B). More specifically, the tumoral mass in CNF1-injected animals was approximatively 30% of the volume measured in vehicle-treated controls. No decrease in tumor size has been detected in mice treated with mCNF1 (One way ANOVA, p = 0.746; Figure 3D).

### Late TMZ treatment is not able to prevent functional deterioration of motor capabilities in glioma-bearing mice

**Figure 5:**
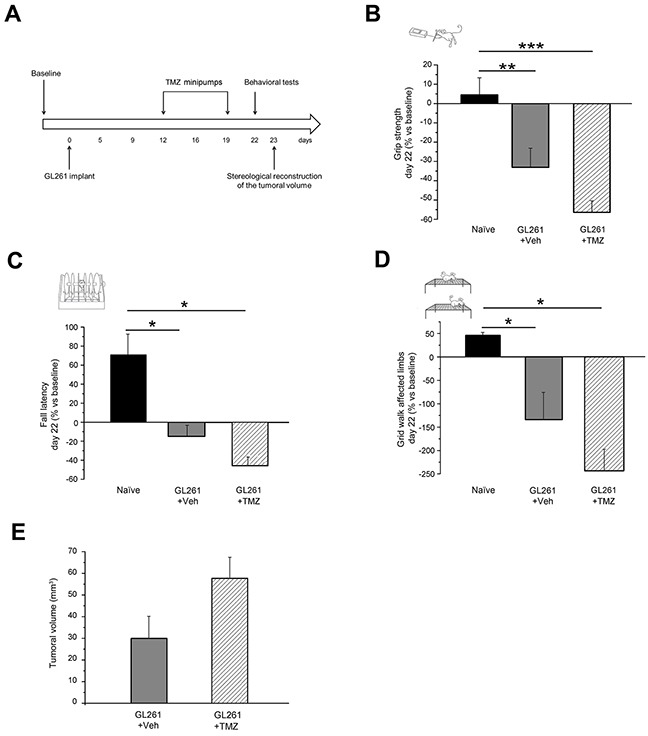
No effect of late TMZ treatment on motor capabilities of glioma-bearing mice **A**. Experimental protocol. **B**. Grip strength of TMZ glioma-bearing mice (patterned column, n = 14) was significantly lower than in naïve (black colum; One-way ANOVA, post hoc Holm Sidak test, p < 0.001) and comparable with that of Veh-treated animals (grey column; p = 0.06). **C**. Fall latency of TMZ glioma-bearing mice was significantly shorter with respect to naïve animals (One-way ANOVA on ranks, post hoc Dunn's test, p < 0.05) and comparable to Veh-treated mice (p > 0.05). **D**. The number of errors performed with affected limbs in the grid walk was not reduced in TMZ as compared to vehicle glioma-bearing mice (One-way ANOVA on ranks, post hoc Dunn's test, p > 0.05). **E**. Stereological reconstruction revealed that late TMZ treatment did not reduce tumor volume (t-test, p = 0.068 vs. Veh group). Data are expressed as mean ± SEM. * p<0.05, ** p< 0.01, *** p <0.001.

We next compared the protective effects of CNF1 with those of classical chemotherapy via temozolomide (TMZ) treatment. To ensure comparability of the two experimental conditions we used direct brain infusion of TMZ for one week via osmotic minipumps [8] implanted at day 12. As for CNF1, the behavioral phenotype of both TMZ-treated animals was analyzed 22 days after GL261 cells injection using grip strength, rotarod and grid walk tests. We found that the performance of TMZ-treated mice (TMZ, n = 14) was not different with respect to vehicle-treated animals both in the grip strength test (One-way ANOVA, post hoc Holm-Sidak method, p = 0.059, Figure 5B) and in the rotarod task (One-way ANOVA on ranks, post hoc Dunn's method, Figure 5C). In addition, the number of errors made with affected limbs in the grid walk test was comparable to that measured in Veh-treated, glioma-bearing mice (One-way ANOVA on ranks, post hoc Dunn's method, Figure 5D). No decrease in tumor size has been detected in mice treated with TMZ with respect to vehicle-treated animals (One way ANOVA, p = 0.068; Figure 5E).

### Molecular mechanisms underlying CNF1 action on peritumoral cells

**Figure 6:**
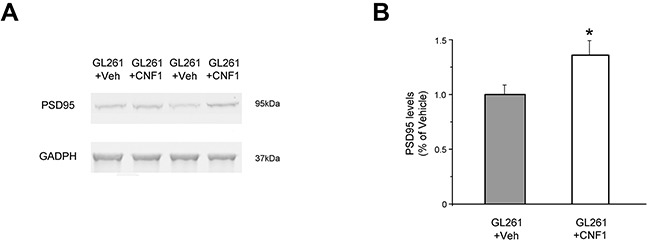
Preservation of functional capabilities in CNF1-treated mice is associated with an increase of PSD95 expression in the peritumoral tissue **A**. Representative immunoblotting of PSD95 expression in CNF1- and Veh-treated animals. GADPH, internal standard. **B**. CNF1 treatment enhances PSD95 protein levels in the peritumoral tissue (t-test, p < 0.05). Data are expressed as mean ± SEM. * p <0.05.

To investigate the molecular mechanisms underlying the long-lasting effects of CNF1 in preserving behavioral functions, we also assessed peritumoral expression of PSD95 in CNF1 and in Veh-treated animals. PSD95 is a postsynaptic marker of excitatory synapses that plays a crucial role in the organization and maintenance of synaptic structure *in vivo* [16]. We measured PSD95 peritumoral expression 23 days after tumor induction by Western blot. We found that PSD95 protein level was notably affected by CNF1 treatment: indeed, peritumoral PSD95 expression in CNF1-treated animals was significantly higher than in control mice (t test, p < 0.05; Figure 6A, 6B).

## DISCUSSION

The main purpose of this study was twofold: (i) to develop sensitive behavioral readouts to detect early motor deficits and longitudinally characterize neural dysfunction in glioma-bearing mice; (ii) to evaluate the therapeutic efficacy of CNF1 administered within a temporal window of translational value, i.e. after the onset of neurological deficits. In order to address these issues, we employed the well-accepted syngenic GL261 model which reproduces many of the histopathological features of human glioma [10] and has been frequently used for preclinical testing in the GBM research [8–10, 17–21].

Using a battery of behavioral tests, we have longitudinally assessed the time course and the extent of motor dysfunction induced by the progression of glioma growth in the motor cortex. Tests employed (i) are easy to carry out, (ii) are repeatable in the same animals on different sessions, (iii) require no particular training and (iv) allow to quantify different aspects of animals’ motor capabilities including strength, dexterity and coordination [5]. So far, animal models of glioma have been studied only at the histological, molecular and pathological levels. Thus, these data allow a more detailed understanding of how tumor growth reverberates on the behavioral functions of glioma-bearing mice, providing normative data for the study of therapeutic strategies for glioma treatment.

More specifically, the grip strength test revealed a very early onset of functional deficit associated to the glioma mass growth, with a significant forelimb weakness appearing 9 days after tumor inoculation. Instead, in the rotarod and in the grid walk tasks, glioma-bearing mice showed a later significant deficit. It is likely that the initial phases of tumor growth do not strongly compromise animals’ motor coordination and dexterity in tasks engaging all the four limbs, while forelimb muscular strength is more sensitive to the deterioration of neuronal function. Consistently, several patients with either high-grade gliomas or low-grade tumors in the primary motor cortex display precocious muscular weakness [22, 23]. It is worth noting, however, that, in the rotarod and the grid walk tasks, glioma growth interrupts the training-dependent improvement of motor performance respectively 12 and 16 days after tumor induction, indicating a first deterioration of motor performance. In summary, behavioral characterization of this mouse model allowed us to identify the appearance of the first symptoms associated with neuronal dysfunction 9-12 days after GL261 cell injection, when the tumor has a relatively small volume, while at day 22 glioma-bearing mice displayed a severe behavioral impairment affecting all features of motor function, in parallel with a dramatic extension of the tumoral mass. It is relevant to note that these tests capture neuronal alterations well before a decline in general fitness (as measured by body weight) in glioma-bearing animals.

These data allowed us to study the efficacy of CNF1 treatment within a clinically relevant therapeutic window. Specifically, we tested whether treating glioma-bearing mice with CNF1 at symptomatic stages was effective in halting tumor growth progression and in protecting neuronal cells from functional deficits. We found that long-lasting Rho GTPase activation starting from 12 days after glioma induction turned out to be successful in stabilizing/reducing glioma volume and the associated physiological deficits. In contrast, TMZ treatment at symptomatic stage was ineffective in preserving motor performance, indicating that TMZ-based therapy does not contribute to preserving animals’ functional capabilities as CNF1 does. Consistently, it has been previously reported that health-related quality of life in patients with glioblastoma is not improved by the addition of TMZ during and after radiotherapy [24]. Since the same batch of TMZ slightly increased animal survival when given early in disease progression [8], however, it is possible that the variability in tumor growth in individual animals might have obscured a small effect of TMZ administration at late stages. Altogether, the present findings are critical for translatability of CNF1 treatment to the human condition, where neurological alterations are typically the first evident signs of the disease progression.

How could the regulation of Rho GTPases by CNF1 translate in the slowdown of tumor growth progression and in the preservation of neuronal functions? We recently demonstrated that CNF1 induces senescence and death of glioma cells. Indeed, a significant downregulation of several trophic factors and enhanced expression of p21/p16 genes were observed in murine and human cell lines [8, 9], indicating the molecular pathways involved in the anti-neoplastic action of CNF1. In addition, the long-lasting activation of Rho GTPases exerts a neuroplastic function on peritumoral neurons, leading to the preservation of structural and physiological properties of cortical networks [9, 15, 25]. Accordingly, in the grid walk task we found that the recovery of motor function of affected limbs in CNF1-treated animals is accompanied by a slight improvement of the performance with unaffected limbs. Since CNF1 promotes activity-dependent plasticity in the adult cortex [15], the toxin might enhance the spontaneous remapping of motor maps in peritumoral areas [26, 27] thus leading to greater functional preservation. Moreover, CNF1 infusion induced a significant increase of PSD95 protein levels in the peritumoral area. This could be due to either an enhanced survival of neuronal cells in the vicinity of tumor, or to an increased density of dendritic protrusions that has been previously documented in CNF1-treated samples [15].

These effects are likely at the basis of the almost complete functional rescue of motor function despite the presence of a glioma mass in CNF1-treated mice. We recently showed, however, that CNF1 treatment is also effective in recruiting astrocytes and microglia/macrophages to the peritumoral tissue of glioma-bearing mice [9], suggesting that CNF1 offers the unique opportunity to impact on glioma-induced brain dysfunctions at multiple sites of action, both by allowing functional and structural plasticity of neurons, and by stimulating a protective action of glial cells.

In summary, these data indicate that CNF1 could be considered a promising strategy for the development of a more effective glioma therapy.

## MATERIALS AND METHODS

All experimental procedures were performed in conformity to the European Communities Council Directive 86/609/EEC and were approved by the Italian Ministry of Health. Adult (>P60) C57BL/6J mice were used in this study (Charles River Laboratories). We used both male and female mice. Females and males were equally distributed among experimental groups. See Supplementary Data for extended methods.

### GL261 glioma cell cultures

The murine glioma GL261 cell line [28] was a kind gift from Dr. C. Sala (CNR Neuroscience Institute, Milan). GL261 cells were aliquotated and stored at -80°C. A new aliquot was defrosted for each experiment. GL261 cells have been tested by analyzing the *in vivo* tumoral growth in naïve animals.

### Tumor induction

Tumor induction was performed as previously described [8, 9]. Briefly, mice received a stereotaxically guided injection of 40,000 GL261 cells (20,000 cells/1 μl Tris HCl solution) into the primary motor cortex [11, 29, 30] using a Hamilton syringe. The GL261 cell solution was slowly delivered at a depth of 0.8–0.9 mm from the pial surface. Sham animals were subjected to the same procedure and infused with vehicle solution.

### Behavioral testing

Mice were tested in three different motor behavioral tests, i.e. grip strength, rotarod and grid walk. For the characterization of the motor GBM model the experimental protocol involved a baseline (day 0) and six further measurements: 5, 9, 12, 16, 19 and 22 days, respectively, after tumor induction. To assess the effects of CNF1 treatment, animals were subjected to the same analysis at day 0 and day 22. To evaluate how the motor behavior changed after the tumor induction, the result of each test obtained in the various days was normalized to the baseline performance. In the grip strength test a peak amplifier automatically measures the peak pull-force achieved by animals’ forelimbs [5]. The rotarod test was performed as described in [5, 31]: mice were placed on a drum, whose rotation speed increased linearly from 4 to 40 rpm. The time spent on the drum was recorded. In the grid walk task mice were placed on a wire mesh for 5 min and the number of correct and wrong steps was calculated [13].

### Drug administration

Twelve days after tumor induction, mice were randomly divided into four groups. The first group received CNF1 injection, the second Tris-HCl buffer injection (control vehicle condition), the third mCNF1 injection [14] and the fourth TMZ minipump implants [8]. Stereotaxic infusions of CNF1 (1 μl of a 80 nM solution), mCNF1 (1 μl of a 80 nM solution) and Tris-HCl (Veh) were made in three separate sites of the primary motor cortex (the site of GL261 cells injection and 0.5 mm medial and lateral to the site of tumor implantation). CNF1/mCNF1/Tris-HCl was slowly delivered at a depth of 0.7–0.8 mm from the pial surface. In a different group of animals, an osmotic minipump filled with TMZ was implanted in the motor cortex ipsilateral to GL261 injection (see Supplementary Data for further details).

### Neuroanatomical analyses

Coronal brain sections (45 μm) were cut on a microtome [9, 31–34]. Serial sections (one of six, spacing factor) were stained with Hoechst dye (1: 500, Sigma). The stereological reconstruction of the glioma volume, was performed using a Zeiss microscope and the Stereo Investigator software.

### Western blot

After decapitation, brains were rapidly removed and the peritumoral tissue was dissected and frozen on dry ice. Proteins were extracted, separated by electrophoresis and blotted; filters were incubated overnight at 4°C with anti-PSD95 and anti-GAPDH primary antibodies (1:1000, Cell signaling Technology; 1:40,000, Abcam). After incubation with infrared labelled secondary antibodies IRDye 700 or 800 (1:20,000 dilution, Li-Cor Biosciences), filters were scanned using an Odyssey® IR scanner and densi-tometry analysis was performed with Odyssey® imaging software 3.1.

### Statistical analysis

All statistical analyses were performed using the SigmaStat Software. Normality of distributions was assessed with Kolmogorov-Smirnov test. The level of significance was p < 0.05.

## SUPPLEMENTARY MATERIALS FIGURES


