# Supplementary material for: Progression of motor deficits in glioma-bearing mice: impact of CNF1 therapy at symptomatic stages
Source: Oncotarget. 2017 Feb 15;8(14):23539–50. doi: 10.18632/oncotarget.15328 (PMC5410325; doi:10.18632/oncotarget.15328)
Supplement: Supplementary file 1 [file oncotarget-08-23539-s001.pdf]

Progression of motor deficits in glioma-bearing mice: impact of CNF1 therapy at symptomatic stages

SUPPLEMENTARY MATERIAL

MATERIALS AND METHODS

Animals

Adult (age > postnatal day 60) C57BL/6J mice were used for this study. Animals were bred in our animal facility and housed in a 12 hours light/dark cycle, with food and water available *ad libitum*. All experimental procedures were performed in conformity to the European Communities Council Directive 86/609/EEC and were approved by the Italian Ministry of Health.

GL261 cells

The murine glioma GL261 cell line was a kind gift from Dr. C. Sala (CNR Neuroscience Institute, Milan). GL261 cells were grown in complete Dulbecco's modified Eagle's medium (DMEM) containing 10% Newborn calf serum, 4.5 g/L glucose, 2 mM glutamine, 100 UI/ml penicillin and 100 mg/ml streptomycin at 37°C in 5% CO2 with media changes three times per week.

Tumor induction

Under avertin anesthesia (intraperitoneal injection of 2,2,2-tribromoethanol solution; 250 mg/kg body weight), C57BL/6 mice received a stereotaxically guided injection of 40,000 GL261 cells (20,000 cells/1 µl Tris HCl solution) into the primary motor cortex (more specifically at the level of cortical forelimb representation; coordinates: 1.75 mm lateral and 0.5 mm anterior to bregma), according to [11] using a Hamilton syringe. Injections were made randomly into either the left or the right brain hemisphere. The GL261 cell solution was slowly delivered at a depth of 0.8–0.9 mm from the pial surface. Body temperature was monitored with a rectal probe and maintained at 37.0°C with a thermostated electric blanket during the surgery. An oxygen mask was placed in front of the animal mouth to facilitate breathing. To prevent dehydration, a subcutaneous injection of saline (0.9% NaCl, 1ml) was delivered at the end of the procedure.

Behavioral tests

Mice were tested in three different motor behavioral tasks, i. e., grip strength, rotarod and grid walk. First, we compared glioma-bearing mice with control naïve animals in order to characterize the motor GBM model. Each animal performed all the tests before GL261 injection (baseline measurement) and 5, 9, 12, 16, 19 and 22 days after tumor implant. For the rotarod test, mice have been subjected to two different baseline tests, because the learning component strongly influences the behavioral output of this task. In addition, grip strength task was also performed in mice receiving a simulated GL261 cells injection (sham animals) to evaluate whether the effects of surgery on motor performance. To exclude any influence of circadian rhythms on behavior, all tests were performed during the same time interval each day (2:00– 5:00 pm; light phase). All the behavioral tests and analysis were performed blind to experimental condition. Mice were weighed at the end of each behavioral session.

Grip strength test

The animal was placed over a base plate, in front of a grasping bar (trapezoid-shaped) whose height is adjustable. The bar is fitted to a force transducer connected to the Peak Amplifier (Ugo Basile S.R.L). When pulled by the tail, the animal grasps at the bar (rodents instinctively grasp anything they can to try to stop this involuntary backward movement), until the pulling force overcomes their grip strength. After the animal loses its grip on the grasping bar, the peak amplifier automatically stores the peak pull-force achieved by the forelimbs and shows it on a liquid crystal display. Three trials per day were performed for each animal and their average was calculated. Values obtained were normalized to the baseline.

Rotarod test

Mice were placed on a drum at the baseline speed of 4 rpm (Letica Inst, Spain). During the 10 min observation period, the rotation speed of the drum increased linearly from 4 to 40 rpm. The time spent on the Rotarod was recorded by an automated unit, which stopped as the mouse fell. Each trial ended when the mouse fell from the apparatus or when 10 minutes had elapsed. Motor performance was assessed by performing 5 consecutive trials in the same day with an interval of 5 minutes between one trial and the next one. Averaged fall latency was calculated for each animal. The apparatus was cleaned with 10% ethanol between trials to stop the build-up of olfactory cues. Values obtained were normalized to the baseline performance.

Grid walk

Mice walk on a grid (32 cm X 20 cm with 11 mm X 11 mm mesh) on which to walk properly they have to put their paws on the wires; these are considered correct steps. Instead, when one of the limb falls in a hole of the grid a wrong step was calculated. The test took 5 minutes for each animal and it was filmed by a camera positioned in front of the grid. A mirror placed below the grid helps to understand which of the limbs made the mistake. The video analysis was made by using a reproduction *frame by frame*. Scoring was done separately for affected (i.e. contralateral to GL261 injection) and unaffected (ipsilateral) limbs. In naïve animals, affected and unaffected limbs were identified with respect to the injection site in the corresponding group of glioma-bearing mice.

The following parameters were calculated:

1. affected limb wrong steps % = affected limb wrong steps/ (affected limb wrong steps + unaffected limb wrong steps) * 100
2. unaffected limb wrong steps % = unaffected limb wrong steps/ (affected limb wrong steps + unaffected limb wrong steps) * 100

Furthermore, we evaluated the asymmetry index by subtracting the number of errors made with unaffected limbs to the number of errors perfomed with affected limbs. A positive asymmetry index indicates a higher number of errors made with the affected limbs. Finally, we also evaluated the number of total steps, that is the sum of correct and wrong steps made with all four limbs. For each measure, values obtained were normalized to the baseline.

Drug administration

CNF1 was obtained from the 39S ISS strain (a generous gift of V. Falbo, Rome, Italy) and purified as previously described (Falzano et al., 1993). The levels of lipopolysaccharide (LPS) in the CNF1 preparation were assessed by the Limulus Amebocyte Lysate (LAL) kinetic chromogenic assay (performed by LONZA Verviers Sprl). The LPS concentration determined by the assay (0.07 ng/ml) was much lower than that required to achieve biological effects (e.g. 1 ng/ml in macrophages, one of the most sensitive cells to LPS). CNF1 C866S (mCNF1), a recombinant CNF1 in which a change of cysteine with serine at position 866 completely abrogated the enzymatic activity on Rho GTPases [14], was used as a control. mCNF1 was a kind gift from E. Lemichez (Inserm, Nice, France), and the recombinant toxin was purified as described previously (Falzano et al., 1993). Twelve days after tumor induction, mice were randomly divided in four groups. The first group received CNF1 injection, the second Tris-HCl buffer injection (control vehicle condition), the third mCNF1 injection and the fourth TMZ minipump implants. Stereotaxic infusions of CNF1 (1 µl of a 80 nM solution), mCNF1 (1µl of a 80 nM solution) and Tris-HCl (Veh) were made into the primary motor cortex mice under avertin anesthesia (intraperitoneal injection of 2,2,2-tribromoethanol solution; 250 mg/kg body weight). Injections were performed in three separate sites: the same site of GL261 cells injection and 0.5 mm medial and lateral to the site of GL261 cells injection. CNF1/mCNF1/Tris-HCl was slowly delivered at a depth of 0.7–0.8 mm from the pial surface. TMZ (Sigma) was dissolved in 100% DMSO at a concentration of 14 mM and then further diluted to 140 µM [8] in a solution of 1% DMSO and 99% Tris-HCl. Under anesthesia (see above), an osmotic minipump (model 2002; Alzet, Palo Alto, CA), connected via PE tubing to a stainless steel cannula (30 gauge), was implanted in the motor cortex ipsilateral to GL261 injection. The minipump was positioned subcutaneously under the neck and the cannula was secured to the skull with acrylic cement. Infusion of TMZ with osmotic minipumps (flow rate, 0.5 µl/h) lasted for one week.

Neuroanatomical analyses and stereological reconstruction of tumor volume

Animals were deeply anesthetized with chloral hydrate and perfused transcardially with PBS 1x followed by fixative (4% paraformaldehyde, 0.1 M sodium phosphate, pH 7.4; PB) 23 days after the tumor induction. Brains were gently removed, post-fixed for 4 h in the same fixative at 4°C, cryoprotected by immersion in 30% sucrose with 0.01% sodium azide solution at 4°C and frozen by isopentane. Coronal sections (45 µm) were cut on a microtome and collected in PBS. Serial sections (one of six, spacing factor) were stained with Hoechst dye (1: 500, Sigma) for the evaluation of glioma volume. Tumor volume was measured in coronal sections using a Zeiss microscope and the Stereo Investigator software (MicroBrightField). In each of the selected slices, with a magnification of 10X, the area of the tumor was delineated. The tumor volume was calculated by summing the measured areas and multiplying by the spacing factor and by the thickness of the slice (45 µm).

Western blot

In order to avoid circadian effects, all animals (CNF1: n = 8; Veh: n = 7) were sacrificed during the same time interval each day (10:00–12:00 h; light phase). Samples were taken from animals that were not previously used for behavioral experiments. After decapitation, brains were rapidly removed and the peritumoral tissue was dissected and frozen on dry ice. Proteins were extracted with lysis buffer (20 mM Tris-HCl pH 7.45, 150 mM NaCl, 10 mM EDTA, 0.1 mM Na3VO4, 1 mM PMSF, 1 µg/ml leupeptin, 1 µg/ml aprotinin, 1% Triton X-100 and

10% glycerol), and the total concentration of the samples was assessed with a protein assay kit (Bio-Rad) using a bovine serum albumin-based standard curve. Protein extracts (30 µg) were separated by electrophoresis and blotted; filters were blocked and incubated overnight at 4°C with anti-PSD95 primary antibodies (1:1000, Cell Signaling Technology). Filters were also probed with anti-GAPDH antibody (1:40,000, Fitzgerald) as an internal standard for protein quantification. Blots were then rinsed in Tween buffered saline (TPBS), incubated in infrared labelled secondary antibodies IRDye 700 or 800 (1:20,000 dilution, Li-Cor Biosciences), washed in TPBS and briefly rinsed in PBS. Filters were scanned using an Odyssey® IR scanner and densitometry analysis was performed with Odyssey® imaging software 3.1. Antibody signal was calculated as integrated intensity of the region defined around the band of interest. Protein amount was evaluated measuring the signal of the band of interest and dividing it by the signal of GAPDH band on the same filter.

Statistical analysis

All statistical analyses were performed using the SigmaStat Software. Differences between two groups were assessed with a two-tailed t test or a Mann-Whitney rank sum test when the two data sets were not normally distributed or had unequal variances. The significance of factorial effects and differences between more than two groups were evaluated with ANOVA/repeated measures (RM) ANOVA followed by Holm-Sidak test for data normally distributed, and with ANOVA on ranks with Dunn's post hoc test for data not normally distributed. Normality of distributions was assessed with Kolmogorov-Smirnov test. The level of significance was $p < 0.05$.

SUPPLEMENTARY REFERENCE

1. Falzano L, Fiorentini C, Donelli G, Michel E, Kocks C, Cossart P, Cabanié L, Oswald E, Boquet P. Induction of phagocytic behaviour in human epithelial cells by Escherichia coli cytotoxic necrotizing factor type 1. Mol Microbiol 1993; 9: 1247-1254.

SUPPLEMENTARY FIGURES

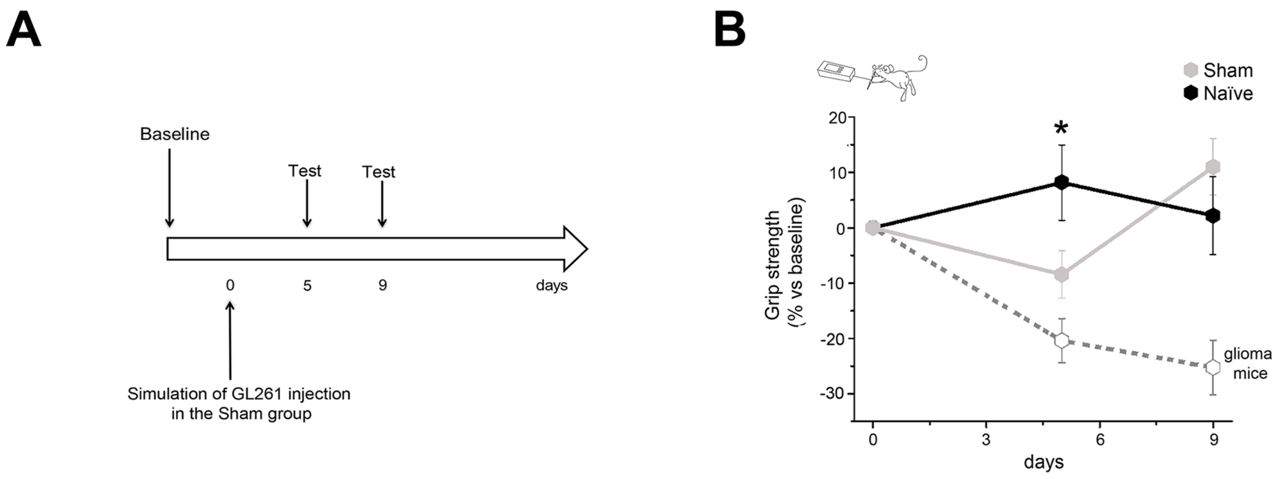

Supplementary Figure 1: Grip strength performance in sham and naïve mice. A. Experimental protocol used to compare sham and naïve mice in the grip strength test. Both groups were evaluated 5 and 9 days after tumor implantation. **B.** A significant decrease of grip strength was found in sham mice (grey line, n = 12) 5 days after tumor implantation (Two-way RM ANOVA, post hoc Holm Sidak method $p < 0.05$), while no difference between the two groups was found at day 9 (Two-way RM ANOVA, post hoc Holm Sidak method $p = 0.231$). Open symbols and dotted line indicate the data recorded in glioma-bearing mice for comparison. Data are expressed as mean ± SEM. * $p < 0.05$.

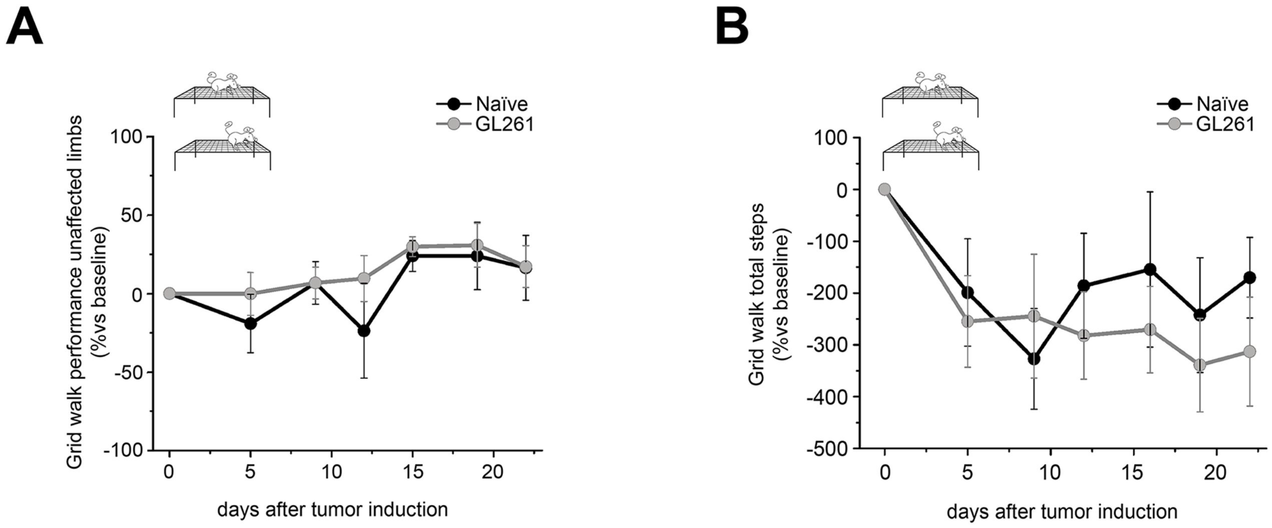

Supplementary Figure 2: Grid walk test in glioma-bearing mice. No significant difference was found in both the number of errors performed with unaffected limbs **A.** and the number of total steps **B.** between naïve (black line, n = 11) and glioma-bearing animals (grey line, n = 9; Two-way RM ANOVA on rank transformed data p = 0. 969 and p = 0.564, respectively). Data are expressed as mean ± SEM.

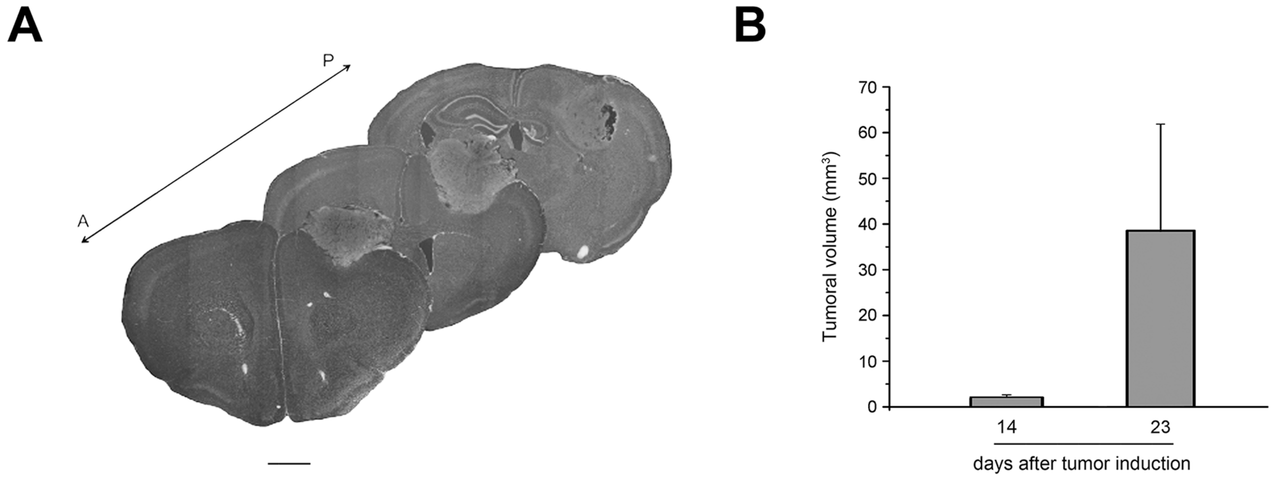

Supplementary Figure 3: Tumor growth in primary motor cortex. A. Representative images from a glioma-bearing mouse 23 days after GL261 cells implantation. White arrows indicate the glioma mass. A, anterior; P, posterior. Scale bar = 1 mm. **B.** Glioma volume quantification 14 (n = 6) and 23 (n = 13) days after GL261 cells injection. A remarkable increase of tumor size was observed between the two time points. Data are expressed as mean ± SEM.

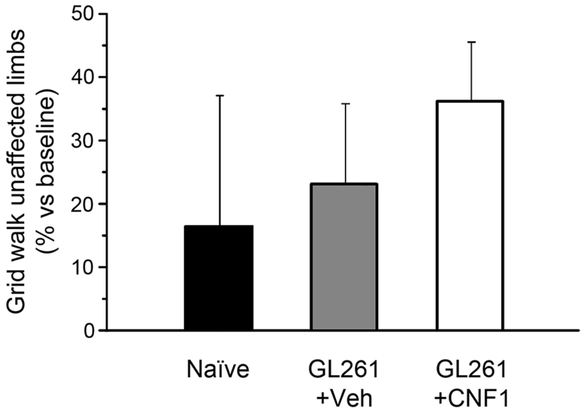

Supplementary Figure 4: Grid walk performance for unaffected limbs in CNF1 glioma-bearing mice. No significant difference among naïve controls (black column), Veh (grey column) and CNF1 (white column) glioma-bearing mice was found in the grid walk performance with unaffected limbs (One-way ANOVA on ranks $p = 0.649$). Data are expressed as mean ± SEM.
